# Three-Year Follow-Up Study Exploring Metacognition and Function in Individuals With First Episode Psychosis

**DOI:** 10.3389/fpsyt.2019.00182

**Published:** 2019-04-12

**Authors:** Abigail C. Wright, Geoff Davies, David Fowler, Kathryn Greenwood

**Affiliations:** ^1^School of Psychology, University of Sussex, Brighton, United Kingdom; ^2^Sussex Partnership NHS Foundation Trust, Worthing, United Kingdom; ^3^Center of Excellence for Psychosocial and Systemic Research, Massachusetts General Hospital, Boston, MA, United States; ^4^Faculty of Health and Medical Sciences, University of Surrey, Guildford, United Kingdom

**Keywords:** first episode psychosis, metacognition, functioning, longitudinal, cognition, negative symptoms, functional capacity

## Abstract

**Introduction:** Research has demonstrated that functional outcome in psychosis is predicted by factors such as neurocognition, functional capacity, symptoms and, more recently, metacognition. Metacognitive ability has been demonstrated to mediate between neurocognition and functional outcome in First Episode Psychosis (FEP). Whether metacognition also predicts longer-term recovery in first episode psychosis is unknown. This study assessed whether neurocognition, functional capacity and metacognitive ability in FEP predicted functional outcome three years later.

**Methods:** Eighty individuals with First Episode Psychosis were re-contacted after an average 3 years (range: 26–45 month follow-up) from baseline. Twenty-six participants (33%) completed completed measures of neurocognition, metacognition, functional capacity, functional outcome (hours spent in structured activity per week) and psychopathology at baseline and at follow-up.

**Results:** Individual regression analyses demonstrated neurocognition, functional capacity, and metacognitive ability at baseline significantly predicted functional outcome at three years. However, when baseline functional outcome was controlled, only metacognitive ability was a significant predictor of change in functional outcome from baseline to follow-up, *p* < 0.001. This model explained 72% (adjusted *r*^2^ = 0.69) of the variance in functional outcome at follow-up. Negative symptoms did not change the model.

**Discussion:** This study demonstrated that better metacognitive ability significantly predicted improvement in functioning in FEP across a 3-year period. This highlights the potential value of clinical interventions that focus on improving metacognitive ability at first point of illness to maximize recovery.

## Introduction

Clinical recovery can be considered improvement in symptomatology and social/occupational functioning (1, 2). Although clinical recovery after an experience of psychosis was previously considered poor (3), recent research has demonstrated that around 50% of individuals with psychosis had favorable outcomes after long follow-up periods (4–6) and this has also been demonstrated with First Episode Psychosis (FEP) (7, 8). A 10-year follow-up study showed 77% of participants showed at least one period of recovery (defined as sustained symptom remission for at least two years) with 46% symptom-free for at least two years (9). However, for (10) and Robinson et al. (8) only 6.6% and 14% (respectively) met the criteria for full recovery after 1 and 5 year(s) (respectively), suggesting clinical recovery during early stages of illness may be slow. There is clear interest in understanding factors that influence clinical recovery in psychosis, particularly FEP where recovery may be more likely (4, 9). Birchwood et al. (11) named this the critical-period, highlighting the importance of early and targeted interventions to prevent further decline (12–14).

Social and occupational functioning, an aspect of clinical recovery, is a measurable aspect of an individual's specific activities of daily living. Hodgekins et al. (15) suggested assessing functioning using time spent in structured activity per week (4, 16), including employment, education, sports, and leisure. Research has demonstrated that time spent in structured activity is on average 63.5 h in the healthy population, 25.2 h in FEP sample (17), and 19.7 h in a psychosis sample with delayed recovery (15). Importantly, engaging in more hours of activity, e.g., paid work, has been associated with reduced symptoms and improved overall functioning in interventions studies (18, 19).

Research has demonstrated difficulties in functioning across the course of recovery in psychosis and, therefore, the importance of focusing on understanding and improving poor functional outcome. Despite advances in psychological interventions for psychosis, outcome remains poor. There is value in the identification of those with psychosis who are at risk of poor functioning across time, to target interventions to reduce this disability. There are four selected lines of evidence which will be discussed here to suggest factors which predict poor functioning: (i) neurocognition, (ii) functional capacity, (iii) negative symptoms and (iv) metacognition.

Research assessing functional outcome within individuals with psychosis has focused on the influence of neurocognitive difficulties (20–22). The relationship between neurocognition and functional outcome has been demonstrated cross-sectionally (23, 24) and longitudinally in schizophrenia (8), FEP (25, 26) and Ultra-High Risk groups (27). However, studies have demonstrated that predicting those who would have poor outcome with neurocognitive variables is substantially more straight-forward than predicting those who would recover (28, 29). This suggests a more complex relationship with additional factors to be explored.

Functional capacity has also been shown to predict real-world functional outcome within schizophrenia (30, 31) and FEP (32). Neurocognitive ability has been shown to be associated with functional capacity (33–35) and functional capacity has been shown to mediate between neurocognition and functional outcome in schizophrenia (36) and FEP (32). A longitudinal study demonstrated functional capacity predicted real-world functioning in psychosis for those with positive, but not for those with negative symptoms (37). The authors suggested that negative symptoms are distinct and can impact functioning, more so than functional capacity.

Models have highlighted that negative symptoms predict functional outcome in psychosis (38, 39). Specifically, negative community symptoms (e.g., amotivation or social withdrawal), when combined with cognitive deficits, have the largest impact on functioning in schizophrenia (40). Longitudinal studies have demonstrated that lower levels of negative symptoms predicted better outcomes in FEP in a large study of 304 participants (41). When assessing positive and negative symptom trajectories, poorer negative symptom trajectories were associated with poorer social functioning, disorganized symptoms, and schizophrenia diagnosis, compared to positive symptom trajectories which were associated with DUP and substance use (42). However, Alvarez-Jimenez et al. (43) demonstrated that whilst symptom remission predicted functional recovery, negative symptoms had little predictive value for long-term functioning. Studies have noted an overlap in the variance in outcome explained by cognition and negative symptoms (25, 44) and, when taking into consideration the role of cognition, symptoms are shown not to predict functioning cross-sectionally (45) and later longitudinally (46). These studies highlight that functional outcome is the product of a complex array of abilities and symptoms.

Following this research, models in psychosis suggest that neurocognition, functional capacity, and negative symptoms influence functional outcome (33, 47, 48). The path between neurocognition and functioning has been shown to be mediated by functional capacity and cognitive processes (36, 47–49), including defeatist performance beliefs and self-stigma (50) and, most recently, metacognition (32).

Metacognition is considered “thinking about thinking” (51) or the way one thinks about one's experience (52). Metacognition involves forming an integrated representation of oneself, others, and the world and using these representations to implement an effective action strategy to perform or accomplish a task (53). Nelson and Narens (54) outlined a metacognitive model suggesting an object-level which (cognitive processes) and a meta-level (an abstract view of the object-level) which are connected by metacognitive processes. Metacognition may be fractionated and three levels of metacognition have been proposed. Firstly, metacognitive ability: capacity to think about one's own cognitions, emotions and behavior, and to use this reflection to respond to challenges (55, 56). Secondly, metacognitive experience: an online appraisal of one's experience, and thirdly, metacognitive sensitivity: a sub-conscious awareness of performance during a task. The first level, metacognitive ability, measured using Metacognitive Assessment Scale (MAS) (57) or Metacognitive Assessment Interview (MAI) (58), is shown to predict real-life functioning in schizophrenia (59–61). In particular, metacognitive ability is show to be a mediator between neurocognition and functioning in schizophrenia (62) and FEP (32, 63). Metacognitive ability is shown to have a large, key role in functioning in psychosis, although this relationship is also influenced by neurocognition and functional skills.

Whether the relationship between metacognition and functional outcome persists across time is unknown. Intervention studies focusing on improving metacognition have demonstrated an increase in real-world functioning (64–67). Longitudinal studies demonstrated that metacognitive ability, particularly one's ability to use representations of self or other to implement effective strategies, predicted social functioning across a 5-month period in schizophrenia (68) and McLeod et al. (69) demonstrated that metacognitive ability predicted negative symptoms at 12 months in FEP, independent of known factors, e.g., gender, DUP and premorbid academic/social adjustment. However, no study has yet assessed the role of metacognitive ability on functioning over a longer follow-up period; particularly within FEP.

It may be suggested that metacognitive ability enables the use of appropriate skills and abilities to perform a task or challenge. For example, an individual may have poor neurocognitive ability, but if they have appropriate metacognitive ability then they may be able to use their available resources and strategies in order to overcome challenges in the environment. Successful outcome, following the utilization of metacognitive ability, may predict engagement in more activities over that predicted by neurocognition, functional capacity skills and negative symptoms. Lysaker et al. (70) demonstrated that those with schizophrenia and high metacognitive ability display better work performance across 6-months, as those with high metacognitive ability were able to see their conclusions as fallible and were able to learn and adapt to the changing demands of work. Therefore, metacognitive ability may be the key predictor of a change in functional outcome across time.

From this, it is hypothesized that functional outcome in FEP at 3-year follow-up will be predicted by metacognitive ability at baseline, independent of neurocognition, negative symptoms, and functional capacity. It is also hypothesized that a change in functional outcome will be predicted by metacognitive ability at baseline, independent of neurocognition, negative symptoms, and functional capacity.

## Materials and Methods

### Procedure

Ethical and Health Research Authority approval was obtained through Camberwell St. Giles Research Ethics Committee (reference number: 17/LO/0055). All participants provided written informed consent at first entry to the study. Participants who gave consent to be re-contacted were contacted after the 3-year period and provided written informed consent at follow-up.

### Design

This was a longitudinal follow-up study exploring the contribution of metacognitive ability to functional outcome at 3-year follow-up with individuals with First Episode Psychosis. Full details of the study design and ethical approval is provided in the protocol (71). Details of the baseline study are provided in an earlier publication (32).

### Participants

Participants with First Episode Psychosis were recruited, via a convenience sample, from outpatient Early Intervention in Psychosis services in Sussex Partnership NHS Foundation Trust Sussex, UK. All participants had been within Early Intervention Services for at least 3 months before entry into the study. All participants received a diagnosis of First Episode Psychosis (F29) by a psychiatrist at entry to the study. Participants with a primary diagnosis of substance misuse disorder or organic neurological impairment were excluded.

### Measures

#### Metacognitive Ability

This was assessed using the Metacognitive Assessment Interview (MAI) (58), which requires the participant to reflect on a recent difficult interpersonal experience and to answer a series of questions reflecting on this experience. The measure assesses the individual's ability for (i) monitoring, identification of feelings and thoughts, (ii) differentiation, distinguishing between dreams, beliefs or assumptions, (iii) integration, reflection on different mental states and rules governing them, and (iv) decentralization, describing the mental state of the other which is independent of their own view. These four subscales are scored between 0 and 5, depending on spontaneity, use of aids/prompts and the sophistication of the answer. The scores for the sub-domains are averaged to provide one multidimensional score. This measure has demonstrated good inter-rater reliability and internal consistency (α = 0.91 for total metacognition), and construct reliability showing correlations amongst the MAI scales (*r* = 0.62–0.9) (58).

#### Function

##### Functional outcome

Time-Use Survey (TUS) (72) is a structured interview [inter-rater reliability 0.99; (15)] during which participants are asked questions regarding the number of hours spent engaged in specific structured activities for the preceding month (D 15), including hours spent in paid work, voluntary work, educational activity, childcare, sports, leisure, and housework activities. A weekly average was calculated for each activity. Two total scores can be produced: (i) constructive economic activity (CEA) including the total hours per week in employment, education, voluntary work, childcare and housework and chores and (ii) structured activity (SA) including the total hours per week in constructive economic activity, leisure activities, and sports activities. Within this study, we used structured activity as the total score. This measure is able to capture differences across clinical groups (15, 73).

##### Functional capacity

The UCSD Performance-Based Skills Assessment (74) provides a total score for real-life performance skills based on role-play tasks. This measure is divided into five sections: (i) finance, e.g., counting money, (ii) communication, e.g., re-arranging a medical appointment, (iii) comprehension/planning, e.g., planning a visit to a theme park, (iv) transport, e.g., reading a bus timetable, and (v) household, e.g., creating a shopping list from a recipe. During each role-play the individual is given points by the researcher from the manual guidelines. These raw scores are totaled for each domain, converted into 0–20 scale then multiplied by 2 and summed to provide a total out of 100. This measure demonstrates high internal consistency (α = 0.88), good validity with other scales (Direct Assessment of Functional Status scale, *r* = 0.86) and good test-retest reliability (*r* = 0.91) (75–77).

#### Neurocognitive Ability

Participants completed a battery of neurocognitive measures, including Verbal and working memory [Logical Memory and Letter-Number Sequencing subscales from the Wechsler Memory Scale (WMS-III)], executive function (Trail-Making Task and Verbal Fluency), Verbal and Performance IQ (Vocabulary and Matrix reasoning tasks). All scores were converted into Z scores using age-scaled population means and standard deviations (78, 78–80). A neurocognitive composite score at baseline was produced from the measures outlined above. IQ was assessed at follow-up using Vocabulary task and Matrix reasoning task (78).

#### Symptoms

Positive and Negative Syndrome Scale (81) was used as this is the most widely used standardized instrument for assessing symptom severity in schizophrenia (82). This measure provided three separate scores for positive, negative and general psychopathology symptoms.

## Planned Analysis

G power estimation was used for a power calculation based on a power of 0.80, effect size of 0.31 (32) and alpha of 0.05. This suggested a total of 44 participants were required when including 4 predictors.

### Hypothesis Testing

Descriptive statistics were produced for neurocognitive ability, metacognitive ability, functional capacity, functional outcome, and symptoms, and scores were compared from baseline to 3-year follow-up using *t*-tests. At baseline, a large battery of neurocognitive measures was collected. At follow-up, only matrix reasoning and vocab measures (as a two-part IQ score) were collected. Therefore, the difference tests (comparing baseline and follow-up) only assessed differences in these measures.

For the main analyses, predictors of functional outcome at three follow-up were explored (predictors included: neurocognition, negative symptoms, functional capacity, and metacognition). In light of the small sample size, and in order to reduce the number of predictors in the model, a series of single regression analyses were used to assess the predictive value of each variable at baseline on functional outcome at 3-year follow-up. After this, a stepwise regression was conducted using only the significant predictors as covariates and metacognitive ability was added to the model in block 2, to assess the independent contribution of metacognitive ability.

Next, the predictive value of variables on change in functional outcome from baseline to follow-up was assessed. The change was assessed by using baseline functional outcome as a covariate, adjusting the mean for the baseline levels, to explore differences in functional outcome at follow-up. Then, significant predictors were used as covariates and metacognitive ability added to the model in block 3. It is important to note that the Time-Use survey has been demonstrated to be sensitive to change over time in clinical trials [see (83, 84)]. Due to the sample size and to minimize the number of predictors within a single model, the independent role of neurocognition, negative symptoms, and functional capacity were used as covariates within three parallel analyses.

## Results

### Data and Assumption Checking

Missing data was considered as “Missing Completely At Random” (MAR), as missing data was not associated with data within the study. All predictor and outcome data were checked for skewness, kurtosis, and outliers. MAI total at baseline displayed a multimodal distribution. There were no significant differences on cognition, functional capacity, functional outcome, symptoms, and metacognitive ability for those who participated in follow-up and those who did not.

### Sample Characteristics

**Figure 1:**
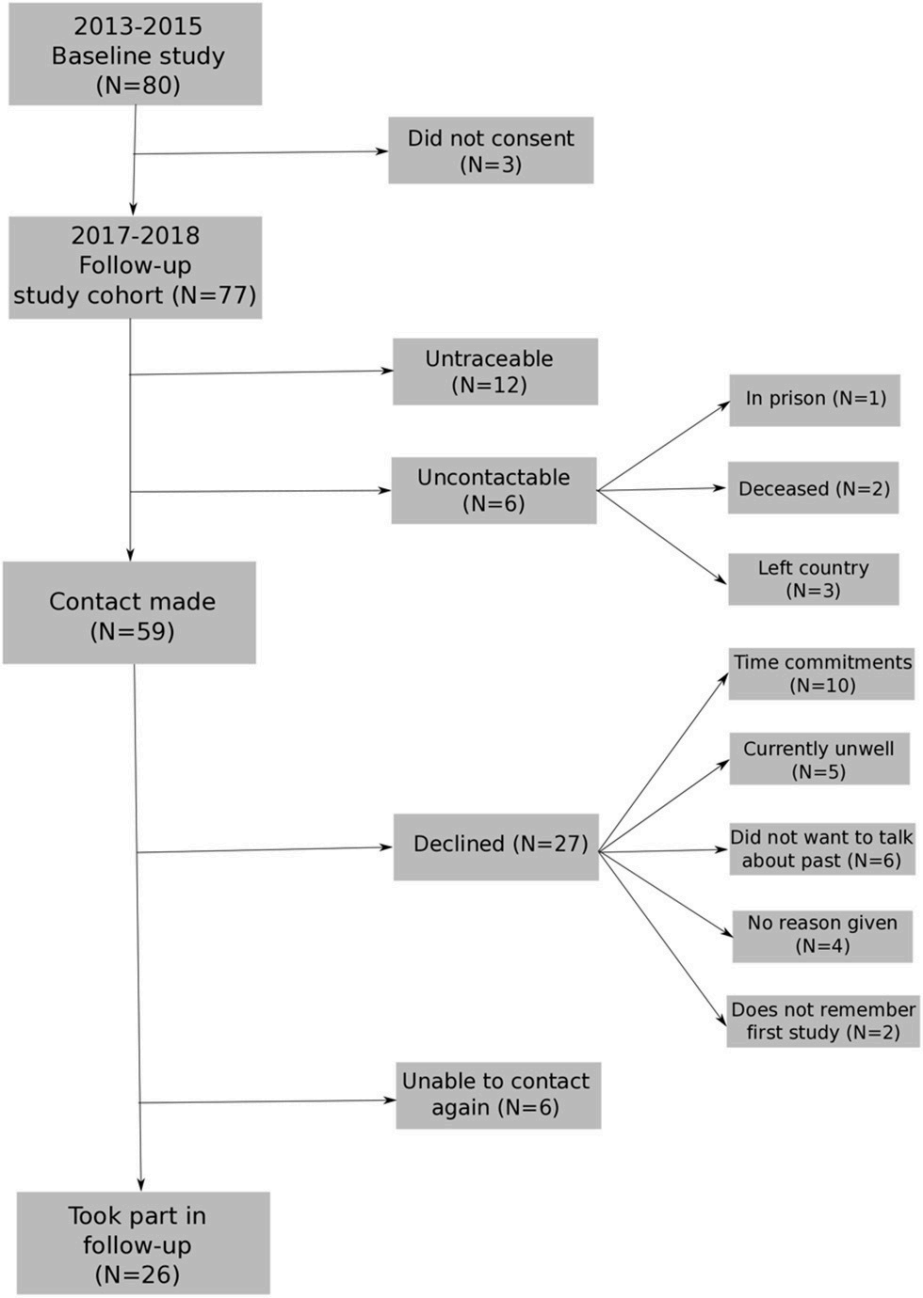
Flowchart for re-recruiting individuals from baseline study into longitudinal study^1^.

The first recruitment phase took place during 2013–2015. The follow-up recruitment phase took place within 2017, after 3 years (average 36-month; range 26–45-month follow-up). The baseline sample included 80 participants with FEP (49 men, 31 female) with a mean age of 26.08 years (*SD* = 5.53). Seventy-seven people provided consent to re-contact. Twenty-six participants from the baseline study took part in the follow-up assessment (see Figure 1 for flowchart of participation). The mean age at follow-up was 28.93 (*SD* = 5.55, range 22–43) with 23 males and 8 females (see Table 1). See Supplement A for distribution of months between baseline and 3 years for the sample followed-up.

**Table 1:** Sample characteristics and descriptive and change statistics for neurocognitive measures, functional capacity, functional outcome, metacognitive ability, and symptoms.

	**Baseline (*N* = 26)**	**Three-year follow-up (*N* = 26)**	**Differences test (baseline to follow-up)**
Age, yrs. (SD)	25.9 (5.94, range 19–39)	29.32 (6.18, range 22–43)	
Gender (M/F)	19/7	19/7	
Education (years)	13.67 (2.09) 13.67 (range 11–16)	13.64 (range 11–16)^a^	
Medication (Y/N)	22/4	18/8	
Matrix reasoning (t-score)^b^	51.6 (8.71) (range 24–68)	54.17 (5.52) (range 41–66)	*t*(23)−1.96, *p* = 0.06
Vocabulary (t-score)	49.62 (12.79) (range 35–70)	54.23 (12.57) (range 21–70)	***t*****(24)−2.2**, ***p*** **=** 0.04*
UPSA total (0–100)	74.66 (13.45) (range 42.94–93.18)	83.09 (9.18) (range 57.96–96.6)	***t*****(24)−4.35**, ***p*** **<** 0.001***
Time Use CEA (hours in activity per week)	22.07 (19.95) (range 0–70.62)	31.66 (22.87) (range 0.81–88.96)	***t*****(25)−2.66**, ***p*** **=** 0.01*
Time Use SA (hours in activity per week)	29.82 (22.3) (3–74.80)	39.31 (24.81) (range 6.06–96.46)	***t*****(25)-2.47**, ***p*** **=** 0.02*
MAI total (0-5)	2.77 (1.32) (range 0.88–4.56)	3.27 (0.87) (range 1.63-4.44)	***t*****(25)-2.28**, ***p*** **=** 0.03*
Symptoms (positive) (7-49)	11.44 (3.63) (range 7–19)	11.87 (4.7) (range 7–23)	*t*(23) −1.23, *p* = 0.23
Symptoms (negative) (7-49)	13.40 (4.06) (range 7–22)	10.87 (3.89) (range 7–26)	***t*****(23)2.92**, ***p*** **=** 0.01**
Symptoms (general) (16-112)	28.68 (6.9) (range 17–43)	26.0 (5.68) (range 18–38)	*t*(23) 1.5, *p* = 0.15
Symptoms (total) (30-201)	53.52 (12.36) (range 32–79)	48.73 (12.84) (range 34–85)	*t*(23) 1.2, *p* = 0.24

Bold: Differences were significant at p < 0.05. *p < 0.05, **p < 0.01, ***p < 0.001. UPSA, UCSD Performance-Based Skills Assessment; CEA, Constructive Economic Activity; SA, Structured Activity; MAI, Metacognitive Assessment Interview (total).

a *Data for follow-up was captured as categories (e.g., GCSE, A-level, Degree, higher degree) which was subsequently converted into years of education to match the baseline groupa*.

b *At baseline, a large battery of neurocognitive measures was collected. At follow-up, only collected matrix reasoning and vocab measures (as a two-part IQ score) was collected. Therefore, the t-tests in this table only assesses differences in these measures*.

### Descriptive Statistics

See Table 1 for descriptive statistics. A number of variables increased over the two timepoints: vocabulary score (*p* = 0.04), functional capacity (UPSA) (*p* < 0.001), functional outcome (time-use structured activity) (*p* = 0.01) and metacognitive ability (MAI) (*p* = 0.03). Negative symptoms decreased (*p* = 0.01) (see Table 1 for descriptive statistics).

### Associations Between Predictor Variables

See Table 2 for correlation matrix for neurocognition, metacognitive ability, symptoms, functional capacity and functional outcome at baseline and follow-up. Age at baseline was significantly associated with functional outcome at follow-up (*r* = 0.4, *p* = 0.027) and included as a covariate in subsequent analyses. For neurocognition, a Confirmatory Factor Analysis was conducted on the z scores of the cognitive variables at baseline and converted into a neurocognitive factor score for each participant.

**Table 2:** Correlation matrix for neurocognition, metacognition, symptoms, functional capacity, and functional outcome at baseline and follow-up.

	**1**	**2**	**3**	**4**	**5**	**6**	**7**	**8**	**9**	**10**	**11**	**12**	**13**
Neurocognitive factor (baseline) 1	–												
Metacognition (baseline) 2	0.61^**^	–											
Positive symptoms (baseline) 3	−0.22	−0.15	–										
Negative symptoms (baseline) 4	0.43^**^	−0.64^**^	0.17	–									
General psychopathology symptoms (baseline) 5	−0.22	−0.39^**^	0.59^**^	0.52^**^	–								
UPSA (baseline) 6	0.48^**^	0.53^**^	−0.09	−0.44^**^	−0.15	–							
Time–use (baseline )7	0.48^**^	0.84^**^	−0.1	−0.49^**^	−0.27^*^	0.39^**^	–						
Metacognition (follow–up) 8	0.66^**^	0.64^**^	−0.33	−0.56^**^	−0.33	0.66^*^	0.4^*^	–					
Positive symptoms (follow–up) 9	−0.32	−0.36	−0.39	0.28	0.17	−0.31	−0.29	−0.59^**^	–				
Negative symptoms (follow–up) 10	−0.54^**^	−0.46^*^	0.3	0.51^*^	0.13	−0.46^*^	−0.34	−0.55^**^	0.71^**^	–			
General psychopathology symptoms (follow–up) 11	−0.28	−0.44^*^	0.38	0.23	0.26	−0.34	−0.47^*^	−0.31	0.75^**^	0.67^**^	–		
UPSA (follow–up) 12	0.71^**^	0.63^**^	−0.29	−0.42^*^	−0.31	0.74^**^	0.3	0.49^**^	−0.29	−0.48^*^	−0.24	–	
Time–use (follow–up) 13	0.29	0.82^**^	0.02	−0.41^*^	−0.23	0.54^**^	0.74^**^	0.42^*^	−0.37^*^	−0.46^*^	0.47^**^	0.5^**^	–

* *p < 0.05*,

** *p < 0.01*.

### Hypothesis 1

In order to test predictors (neurocognition, functional capacity, negative symptoms and metacognition) of functional outcome at 3-year follow-up, individual regression analyses were conducted. Neurocognitive ability at baseline did not significantly predict functioning at 3 years, *p* = 0.24. Functional capacity, *F*_(2, 25)_ 6.66, *p* = 0.01, negative symptoms, *F*_(2, 23)_ 5.69, *p* = 0.01, and metacognitive ability, *F*_(2, 24)_ 27.97, *p* < 0.001, were significant predictors of functioning at 3 years. Including negative symptoms as a covariate in the above analyses did not substantially change the significance levels for neurocognition and metacognitive ability *(p* = 0.44 and *p* < 0.001, respectively). However, when controlling for negative symptoms, functional capacity^1^ at baseline was no longer a significant predictor of functional outcome at follow-up (Δ*R*^2^ = 0.07 *p* = 0.13).

Next, in order to test whether metacognition predicted functional outcome at follow-up independent of other known factors, significant predictors (functional capacity and negative symptoms) were included in the first block of the stepwise regression then the independent contribution of metacognitive ability (MAI) to functioning at three-year follow-up was assessed (see Table 3). This model explained 77.1% (adjusted *r*^2^ = 0.72) of the variance in functional outcome at follow-up *(R* = *0.7*7, *F*_(4, 23)_ 16.01, *p* < 0.001). MAI significantly improved the model (Δ*R*^2^ = 0.35, *p* < 0.001) explaining 34.6% of the 77% (adjusted *r*^2^ = 0.72) total variance explained.

**Table 3:** Full regression model for predictive value of metacognitive ability on functional outcome at three years, whilst controlling for age, UPSA, and PANSS negative symptoms.

		**B**	**SE B**	**β**	***p*-value**	**CI**
**MODEL 2**						
	Constant	7.09	35.2			
	UPSA	−0.58	0.44	−0.33	0.2	−1.5, 0.33
	PANSS negative	−0.14	1.15	−0.02	0.91	−2.54, 2.27
	Age	0.89	0.54	0.22	0.12	−0.25, 2.03
	MAI (total)	18.99	3.54	1.05	< 0.001^***^	11.57, 26.4

*** *p < 0.001*.

*UPSA, UCSD Performance-Based Skills Assessment; PANSS, Positive And Negative Syndrome Scale; MAI, Metacognitive Assessment Interview (total)*.

### Hypothesis 2

In order to assess predictors (neurocognition, functional capacity, negative symptoms, and metacognition) of a change in functional outcome from baseline to 3-year follow-up, individual regression analyses were conducted, controlling for baseline functional outcome. Neither neurocognition (*p* = 0.22), functional capacity (*p* = 0.57) nor negative symptoms (*p* = 0.35) predicted change in functional outcome. Metacognitive ability (MAI) was a significant predictor of change in functional outcome at follow-up, when including baseline functional outcome as a covariate. This model explained 72% (adjusted *r*^2^ = 0.69) of the variance in functional outcome at follow-up (*R* = 0.72, *F*_(3, 25)_ 19.22, *p* < 0.001). MAI significantly improved the model (Δ*R* = 0.12, *p* = 0.01), explaining 12% of the 72% total variance explained (see Table 4). VIF values were inspected to check multicollinearity and the score was acceptable (85, 86). Even when separately controlling for negative symptoms, neurocognition, and functional capacity in parallel analyses, metacognitive ability was still a significant predictor of change in functional outcome from baseline to follow-up [controlling for neurocognition (*p* = 0.005), negative symptoms (*p* = 0.006), and functional capacity (*p* = 0.001)].

**Table 4:** Full regression model for predictive value of metacognitive ability on change in functional outcome from baseline to follow-up, whilst controlling for baseline functional outcome, and age.

		**B**	**SE B**	**β**	***p*-value**	**CI**
**MODEL 2**						
	Constant	−25.69	14.12			
	Age	0.93	0.53	0.21	0.1	−0.18, 2.04
	Time use baseline	0.21	0.24	0.18	0.38^a^	−0.28, 0.70
	Metacognitive ability (MAI)^b^	12.21	4.00	0.61	0.01^**^	3.88, 20.53

** *p < 0.01*.

a *When include age as a covariate, functional outcome at baseline was no longer significant*.

b *MAI, Metacognitive Assessment Interview (total)*.

**Figure 2:**
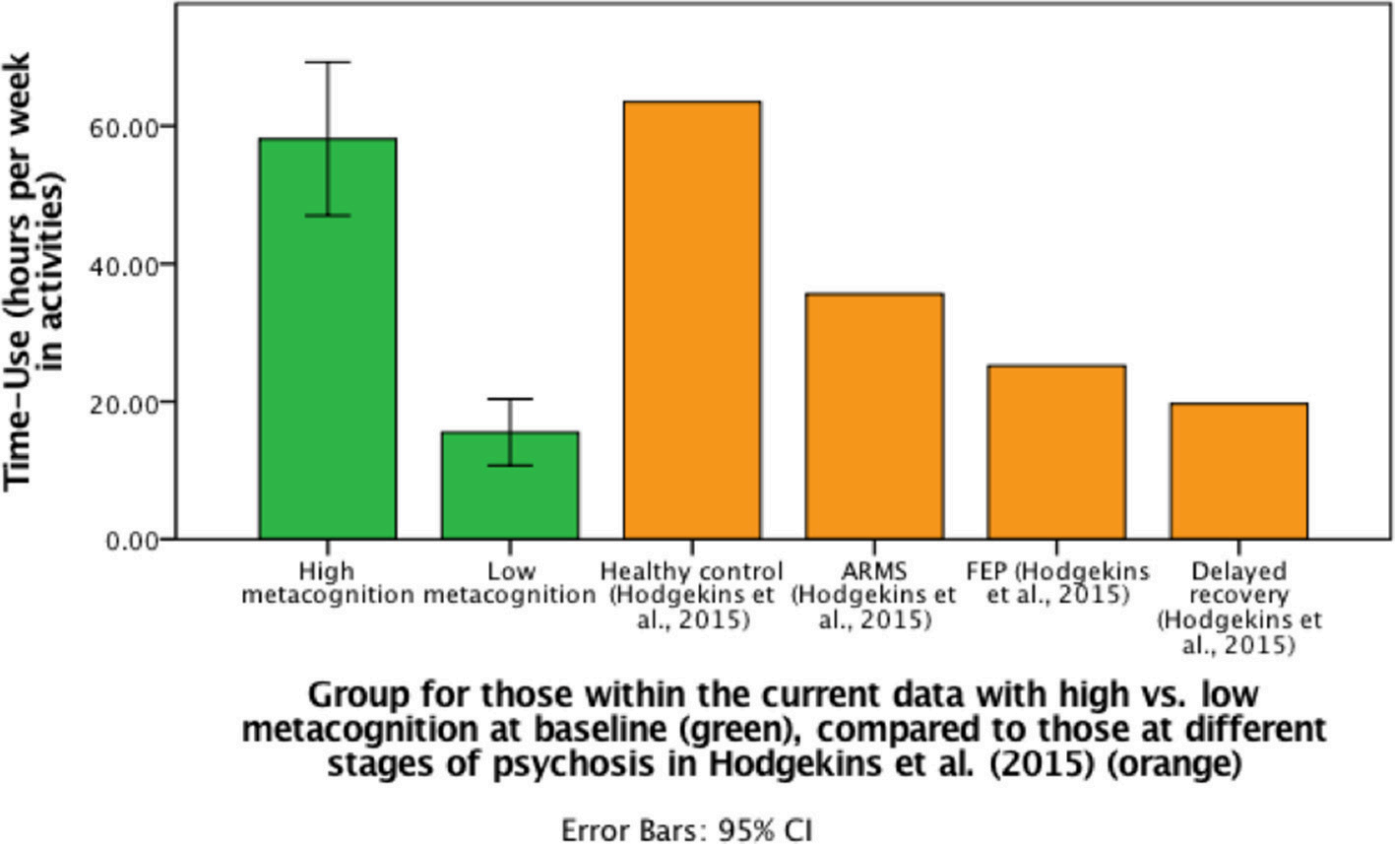
Bar graph to demonstrate differences in mean follow-up time-use scores (including CI for current data) for those with high or low metacognitive ability at baseline compared to previous data from Hodgekins et al. (15).

For those participants who were followed-up, metacognitive ability (MAI) at baseline demonstrated a bivariate distribution (Supplement B). We, therefore, compared those with FEP and either high or low metacognitive ability graphically, with other previous samples. Specifically, we divided participants into two groups: high MAI at baseline (*N* = 14) or low MAI at baseline (*N* = 12), using mean split, to assess the changes in time-use scores between the groups. Individuals in the high MAI group demonstrated a difference in hours spent in structured activity between baseline (*M* = 50.32, *SD* = 23.98) and follow-up (*M* = 58.13, *SD* = 19.29) (*p* = 0.05), but for the low MAI group there was no significant difference (*p* = 0.17) in structured activity between baseline (*M* = 13.17, *SD* = 7.7) and follow-up (*M* = 15.53, *SD* = 7.63) (see Figure 2).

## Discussion

This was the first study to assess the role of metacognitive ability on functional outcome longitudinally across three-years in First Episode Psychosis. This study was able to demonstrate that functional outcome improved over time and, whilst negative symptoms and functional capacity predicted functioning at 3 years, the improvement in functioning was largely predicted by metacognitive ability and baseline functioning.

Within this study, individuals with FEP significantly improved in neuro cognitive ability (vocabulary), real-life functional capacity skills, metacognitive ability and negative symptoms over 3 years. At baseline, individuals within this sample were demonstrating typical mean activity levels for an FEP sample (29.82 h per week) [see (15)], but there was an improvement of 9 hours in structured activity at three-year follow-up, resulting in a time-use score similar to an ARMS group [see (15)]. There was an increase in functional capacity, in that individuals at follow-up were similar to those typically residing independently and employed (76). There was a significant improvement in verbal neuro cognitive ability (vocabulary). Studies in the general population suggest vocabulary is stable over time (87), including studies within schizophrenia (88, 89). The increase in verbal cognition may be the consequence of an initial drop in neuro cognitive ability, particularly verbal IQ (90, 91), which then recovered throughout the follow-up period.

Findings demonstrated that functional capacity and negative symptoms, but not neurocognition, at baseline predicted functional outcome at 3 years after FEP, supporting and furthering research (33, 37, 41, 42). Neurocognition did not directly predict functional outcome at 3-years. However, previous studies have suggested that neurocognitive factors only predict a small amount of variance in real-world functioning (36) and other factors have a more substantial role. Alternatively, neurocognition may have an indirect role, via functional capacity, as the present study demonstrated an association between neurocognition and functional capacity, or neurocognition and negative symptoms as Fervaha et al. (92) demonstrated, using a factor analysis, that negative symptoms may be divided into amotivation and diminished expression and it is the former factor, combined with cognitive deficits, that has the largest impact on functioning in schizophrenia.

Importantly, this study demonstrated that functional outcome at three-years was predicted by metacognitive ability, supporting previous cross-sectional studies (32, 62, 93). Metacognitive ability was also the only significant predictor of improvement in functioning, accounting for a significant change in functioning over time. Therefore, whilst negative symptoms and functional capacity skills predicted functioning at 3-years, this study highlights that those with higher metacognitive ability at baseline may be better able to make use of strategies and resources (e.g., from the early intervention service) to improve their functioning over time, compared to those with lower metacognitive ability who may need more guidance in order to utilize the services available to them.

In further exploring metacognitive ability at baseline, it was evident that individuals who displayed low metacognitive ability at baseline demonstrated limited change in functioning at 3-year follow-up, compared to individuals who displayed adequate metacognitive ability. This may suggest that those with better metacognition were better able to reflect on their thoughts, strengths, as well as perspectives of others, and use appropriate strategies to implement in the real world. Metacognitive ability was a longitudinal predictor of functional outcome, independent of IQ. Whilst the sample size in the group is small, this supports the main hypothesis that early metacognitive factors influence change in functioning.

A large amount of the variance in time-use at follow-up was predicted by baseline time-use and age. Therefore, individuals with better initial functioning are more likely to show an improvement later on, compared to those who had lower functioning who showed no change in already poor functioning. This finding may suggest that those with poor functioning at baseline have poorer metacognitive abilities. The poor metacognitive ability then may predict lower prospective functioning or strategies to improve their poor functioning or are less motivated, due to poor cognitive and metacognitive ability (94, 95), both of which lead to less change or improvement in functioning over time. This study defined functional outcome as a measurable aspect of an individual's specific activities of daily living and social and occupational functioning. The Time-Use Survey is a relatively objective and specific measure of functioning, sensitive to change over time [see (16, 83, 84) and across stages of psychosis see (15)]. Future studies could look at changes in subjective recovery outcomes [see (96)], following the service user movement (97), to identify longitudinal relationships with metacognition. These findings highlight the importance of the Early Interventions services to focus on improving functioning and encouraging early help-seeking to prevent low levels of functioning initially.

These findings can be taken forward in two ways: (i) poor metacognitive ability may be a marker for poor outcome in psychosis later on, and (ii) metacognitive ability may be a key ability for interventions to target in early psychosis to improve subsequent functioning. If metacognitive ability does play a role, metacognitive interventions which have previously demonstrated to be associated with decrease in symptoms (98), may also be useful for improving functional outcome in psychosis (67). Metacognition Reflection and Insight Therapy (MERIT) is specifically aimed at improving metacognitive ability (99). However, De Jong et al. (100) recently demonstrated, in a trial of MERIT for individuals with schizophrenia, evidence of improved metacognitive ability but not functioning. The lack of improvement in functioning may be due to shorter follow-up period or may be accounted for by other factors, e.g., functional capacity or negative symptoms [see (32, 101)]. Therefore, new interventions, such as cognitive remediation, should continue to aim to improve both cognitions and real-life skills and additionally consider training metacognitive ability [e.g., Cella et al. (102)].

### Limitations and Future Studies

Firstly, there was a low follow-up rate. This may be due to the long period between the two assessment points and during this time the participant either moved out of area, lost contact with study team, or could not remember the first study due to length of time or being unwell during the first assessment. A consequence of this low follow-up rate was the small sample size, which limited power and it was not possible to fully explore the role of negative symptoms, alongside metacognitive ability. Future studies using a large sample can confirm the results whilst (i) controlling for all symptoms and (i) exploring the interaction between symptoms and metacognitive ability. Follow-up rate could be improved by continuity of the researcher across research visits, regular contact to maintain rapport, and updating of contact information.

In terms of measurement, this study used the PANSS to assess negative symptoms which may not align with current negative symptoms conceptualizations [see (103)], e.g., negative symptoms are a distinct therapeutic area of interest and negative symptoms are independent from depression, medication side effects and neurocognition. Future studies could assess negative symptoms using Clinical Assessment Interview for Negative Symptoms (CAINS) (104) or Brief Negative Symptom Scale (BNSS) (105) and could assess, or control for, other psychotic symptoms which could be deemed as secondary negative symptoms (40).

This sample was on average lower on symptoms of psychosis at baseline and follow-up (106) compared to other FEP samples (69, 107), which may explain the lack of change in positive symptoms. Whilst this study demonstrated that metacognitive ability, measured using Metacognitive Assessment Interview, had a large predictive role on functioning, other measures of metacognition, e.g., metacognitive experience (online appraisal) has been associated with social, real-world and work functioning (108–110). Future studies should aim to replicate this follow-up study with addition of other metacognitive measures. Finally, age was used as a covariate within the main analyses assessing predictors of outcome, as it was associated with functional outcome at follow-up. Age was recently demonstrated as a positive predictor of structured activity in At Risk Mental State (ARMS) group (111) and it may be suggested that age is a proxy for illness severity as those who have an earlier psychosis onset may have more difficulties in functioning later on (112). However, age of onset is difficult to measure and research suggests that premorbid IQ accounts for this difference (113). Analyses without age as a covariate demonstrated no difference in the results.

## Conclusion

The present 3-year follow-up study was able to demonstrate that metacognitive ability at baseline significantly predicted improvement in functioning after 3 years, in FEP. This was independent of neurocognition, functional capacity, and negative symptoms. This study highlighted the importance of intervening early to enhance metacognitive ability over neurocognitive ability or functional capacity, in order to improve functioning later on, and to target interventions to improve functioning in those with poor metacognitive ability in the early stages of psychosis. Future studies should aim to replicate this within a larger sample.

## Ethics Statement

Ethical and Health Research Authority approval was obtained through Camberwell St. Giles Research Ethics Committee (reference number: 17/LO/0055). All participants provided informed consent at first entry to the study and participants who gave consent to be re-contacted were contacted after the three-year period.

## Author Contributions

AW, KG, and DF developed the hypotheses for the study. GD collected the baseline data and AW collected the follow-up data. AW produced the manuscript with reviewing and editing from all authors (KG, DF, and GD).

### Conflict of Interest Statement

The authors declare that the research was conducted in the absence of any commercial or financial relationships that could be construed as a potential conflict of interest.

## Footnotes

^1^11 people were untraceable: 1 participant was not on the records system and 10 people provided contact details which were now out-of-date and were no longer connected to services.
