# Supplementary material for: Three-Year Follow-Up Study Exploring Metacognition and Function in Individuals With First Episode Psychosis
Source: Front Psychiatry. 2019 Apr 12;10:182. doi: 10.3389/fpsyt.2019.00182 (PMC6473558; doi:10.3389/fpsyt.2019.00182)

**Supplementary data**

**Supplement A**: Bar chart for distribution of months between baseline and three years for full sample.


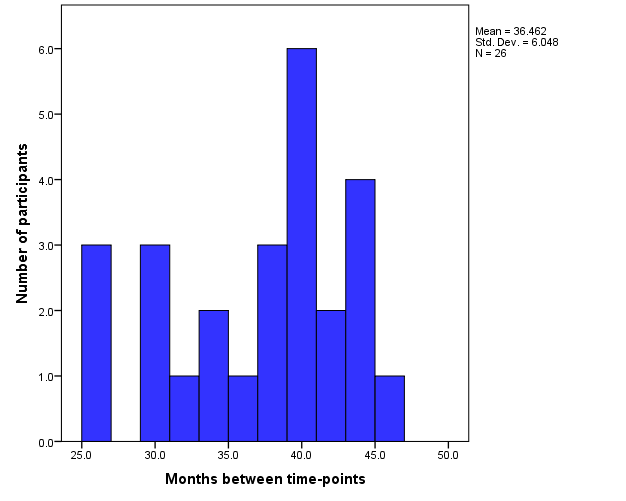


**Supplement B**: Bar chart for distribution of baseline metacognitive ability for the follow-up sample.


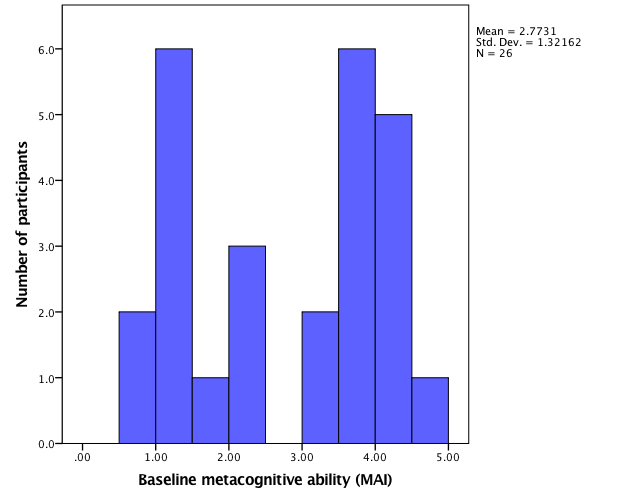

Supplement: Supplementary file 1 [file Data_Sheet_1.docx]
